# Cause and Consequences of Genetic and Epigenetic Alterations in Human Cancer

**DOI:** 10.2174/138920208785699580

**Published:** 2008-09

**Authors:** B Sadikovic, K Al-Romaih, J.A Squire, M Zielenska

**Affiliations:** 1 Department of Pediatric Laboratory Medicine, Hospital for Sick Children, Toronto, Canada;; 2 The Ontario Cancer Institute, Princess Margaret Hospital, Toronto, Canada

**Keywords:** Genetics, epigenetics, DNA methylation, genomic instability, cancer, tumour evolution.

## Abstract

Both genetic and epigenetic changes contribute to development of human cancer. Oncogenomics has primarily focused on understanding the genetic basis of neoplasia, with less emphasis being placed on the role of epigenetics in tumourigenesis. Genomic alterations in cancer vary between the different types and stages, tissues and individuals. Moreover, genomic change ranges from single nucleotide mutations to gross chromosomal aneuploidy; which may or may not be associated with underlying genomic instability. Collectively, genomic alterations result in widespread deregulation of gene expression profiles and the disruption of signalling networks that control proliferation and cellular functions. In addition to changes in DNA and chromosomes, it has become evident that oncogenomic processes can be profoundly influenced by epigenetic mechanisms. DNA methylation is one of the key epigenetic factors involved in regulation of gene expression and genomic stability, and is biologically necessary for the maintenance of many cellular functions. While there has been considerable progress in understanding the impact of genetic and epigenetic mechanisms in tumourigenesis, there has been little consideration of the importance of the interplay between these two processes. In this review we summarize current understanding of the role of genetic and epigenetic alterations in human cancer. In addition we consider the associated interactions of genetic and epigenetic processes in tumour onset and progression. Furthermore, we provide a model of tumourigenesis that addresses the combined impact of both epigenetic and genetic alterations in cancer cells.

## INTRODUCTION

It is well-established that an important part of the cancer etiology lies in stepwise accumulation of genetic changes [1]. DNA mutation leads to aberrant RNA and protein, with widespread deregulation of transcription during oncogenesis. The phenotypic outcomes of genetic changes in cancer have led to a general classification of cancer genes as either tumour suppressors, which are involved in inhibition of cell growth and survival, or oncogenes which promote these effects [2]. Genetic change not only plays a significant role in tumourigenesis, but it is also associated with inter- and intra-tumour heterogeneity [3]. A major challenge facing cancer researchers today is to understand how genomic change can lead to the acquisition of such cellular heterogeneity [4]. New models of oncogenomic progression must consider the *combined effect* of epigenetic and genetic change and concomitant causation of tumour heterogeneity.

The term epigenetics was first introduced by a British embryologist and geneticist Conrad Hal Waddington in 1940, and was used to describe the study of the causal analysis of development [5]. Today, epigenetics refers to the study of heritable changes in gene expression without the change in gene sequence. These heritable changes are propagated as covalent chemical changes to the cytosine bases and are referred to as DNA methylation. Regulation of chromatin compaction and DNA accessibility through spatial and temporal distribution of these chemical signals ensures appropriate genomic responses across different developmental stages and tissue types. In contrast, the deregulation of epigenetic patterns leads to induction and propagation of disease states [6]. The maintenance of these epigenetic signals through cell divisions ensures appropriate regulation of gene activation and repression. DNA methylation uniquely fits the description of an epigenetic mechanism as in addition to playing a role in regulation of gene expression, it is heritable with a clearly defined mechanism of propagation through cell division [7]. In addition to DNA methylation, other mechanisms including histone tail modifications, ATP-dependent chromatin remodelling or non-coding RNAs play an important role in gene regulation and chromatin compaction, but their heritability is less clear. 

The hallmark of cancer is the deregulation of gene expression profiles and disruption of molecular networks [2]. Mutation and genomic instability provide tumours with sufficient diversity, so that cells with adaptive and proliferative advantage can evolve in a Darwinian manner. However, it has become evident that epigenetic factors, particularly heritable changes in DNA methylation, may confer an additional selective advantage to tumours. While there is some understanding of how such genetic and epigenetic changes may influence the gene expression, and thereby tumour evolution, it is less clear how these mechanisms may influence each other, and how these cumulative changes may co-evolve and influence gene expression during tumourigenesis. This review provides an overview of the literature including some recent developments that give insights into the important question of co-evolution of epigenetic changes in tumourigenesis and cancer progression. 

In the first section we review our current understanding of different types of genetic changes in cancer, and provide some specific examples of each. The second section focuses on DNA methylation where we review both normal functions of DNA methylation, disruptions of DNA methylation in human disease, and changes in DNA methylation in human cancer. The final sections focus on literature evidence of combined epigenetic and genetic changes in oncogenes and tumour suppressors, and addresses how DNA methylation may influence genomic stability. We provide an epi/genetic model of tumour evolution and conclude by discussion of its implications in cancer biology. 

## GENETIC CHANGES IN CANCER

Cancer develops as a result of cellular acquisition of specific growth advantages through the stepwise accumulation of genetic and chromosomal changes. Since several genetic alterations are generally required for a cancer to fully develop, the malignant phenotype is determined in part by the combined disruption of tumour suppressor genes and activation of oncogenes. Cancer genomes can be highly unstable and typically exhibit extensive genomic changes, ranging from intragenic mutations, to gross gains and losses of chromosomal material (aneuploidy) [8-10]. In the following section we will review our current understanding of genetic changes underlying the deregulation of tumour suppressor genes and oncogenes while providing some common examples of each (Table **1**), and discuss the correlation of such changes to overall genomic instability.

### Tumour Suppressor Inactivation

Genetic mutations, deletions and allelic loss of tumour suppressor genes during tumourigenesis lead to an aberrant or absent RNA transcript and concomitant loss of the function of the translated protein.

#### Mutations

Intragenic mutations that inactivate genes with roles in maintenance of genomic integrity and various tumour suppressor pathways are common in cancer. These genetic alterations directly or indirectly suppress the normal function of tumour suppressor genes resulting in disruption of cell cycle control and shifting the balance in favor of cell proliferation [11, 12]. One of the most commonly mutated tumour suppressor genes in human cancer is P53. It is localized to chromosome 17p13 and its inactivation is central to the pathogenesis of many tumours including breast cancer, brain tumours, and sarcomas [13, 14]. Inactivation of P53 by mutations is the most frequent and earliest detectable genetic alteration during glioblastoma progression. Glioblastoma is the most common brain tumour where the P53 mutation pattern is characterized by frequent G:C-->A:T mutations at CpG sites which is seen in 60% of the precursor low-grade astrocytomas [15]. Inactivation of P53 in osteosarcoma, the most common cancer of bone in children, can occur by mutations in the gene itself or by alterations of its regulatory genes. Alterations of P53 gene by point mutations is evident in 30 % of osteosarcoma tumours [13, 16]. These mutations can be detected before or after the development of osteosarcoma metastasis, an indication that P53 mutations may be an early event in this disease [17]. Germ-line mutations of P53 lead to Li-Fraumeni syndrome and predispose affected patients to a variety of tumours, particularly sarcomas [18]. 

Another example of the commonly mutated tumour suppressor genes are BRCA1 and BRCA2, which are the only known high penetrance genes involved in breast cancer susceptibility [19, 20]. Most of the mutations that affect these two genes result in protein truncations, which often involve small insertions, deletions or nonsense mutations. About 10% of breast cancer cases that are due to BRCA1 or BRCA2 mutations are considered familial, but the majority of breast cancer cases are sporadic. Only a small fraction of the remaining risk is attributed to germline mutations in other known genes (for example, the P53 tumour suppressor, or the PTEN gene) [21].

Many other examples of mutations of tumour suppressors are evident in cancer and include RB1, APC, PTEN, P21 and others. Some examples and their major tumour sites are listed in (Table **1**).

#### Deletions and Allelic Loss

In addition to mutational inactivation of tumour suppressors which usually involve single nucleotide changes, inactivation of these genes can also be caused by loses of large chromosomal regions or entire chromosomes. Examples of tumour suppressors that are affected by deletions or allelic loss include major regulators of G1 to S transition of cell cycle, the retinoblastoma gene (RB) [22] and the INK4 gene locus [23]. Children with a germline mutation in one of their RB alleles are likely to experience bilateral multifocal retinoblastoma [12]. This gene is located on the long arm of chromosome 13, a region with loss of heterozygosity (LOH) in approximately 60% of osteosarcoma tumours [24, 25]. Gross structural rearrangements of the RB gene are present in up to 30% of osteosarcoma tumours, and point mutations appear to be far less frequent, occurring in less than 10% [25, 26]. Heterozygosity for germ line mutations in RB predisposes patients to the hereditary form of retinoblastoma, and these patients have a significant increase in the frequency of primary and radiation related osteosarcoma [27]. Other studies on sporadic osteosarcoma reported the presence of RB gene alterations in about 70% of the cases and correlated alterations in this gene to late stages of osteosarcoma development [28]. These alterations include structural rearrangements, complete deletions or less frequently point mutations.

The INK4 locus on chromosome 9p21 codes for P16^INK4A^, P15^INK4B^ and P14^ARF^ genes. While P14^ARF^ is involved in the P53 pathway, P16^INK4A^ is a tumour suppressor gene that inhibits CDK4, which in a complex with cyclin D1, facilitates the transition from G1 to S phase in cell cycle by phosphorylating RB. Deletion of INK4 locus was reported in 10% of osteosarcomas [23]. Loss of P16^INK4A^ function results predominantly from INK4A deletions rather than point mutations [23, 28]. Moreover, chromosome region 12q13 is amplified in a subset of osteosarcoma [23]. This region is a genomic location for CDK4 (in addition to MDM2) genes. Higher levels of CDK4 may lead to RB phosphorylation impairing its function in cell cycle control [105]. CDK4 gene is amplified in 9% of osteosarcoma tumours [23] making this event significant in osteosarcoma pathogenesis as it can impact the RB pathway.

Another example of a frequently deleted tumour suppressor is PTEN. This gene is involved in modulation of the phosphatidylinositol-3-kinase (PI3K) pathway and is required for activation of the important regulatory protein kinase AKT. The PTEN locus at chromosome 10q24 is subject to frequent (~40%) genomic deletions in prostate cancers [106], with a significant association between PTEN deletion and an earlier onset of disease recurrence and a greater likelihood of metastatic disease. 

In contrast to the loss of tumour suppressor genes, the process of tumour initiation and progression also requires gains or activation of oncogenes.

### Oncogene Activation

The major types of genetic changes that result in oncogene activation are mutations, chromosomal translocations, or gene amplifications. Oncogene mutations have a gain of function or dominant-acting role in tumourigenesis.

#### Mutations

When an oncogene is activated by a mutation, the structure of the encoded protein is changed in a way that enhances its transforming activity. An example of commonly mutated oncogenes is the RAS oncogene family, which when mutated encode proteins that remain in the active state and continuously transduce signals by linking tyrosine kinases to downstream serine and threonine kinases. These stable signals induce continuous cell growth. Mutations of k-RAS are common in carcinomas of the lung, colon, and pancreas [107], whereas mutations of n-RAS occur principally in acute myelogenous leukaemia and the myelodysplastic syndrome [108].

#### Chromosomal Translocations

Oncogenic translocations involve rearrangements of chromosomes that result in formation of protein coding genes with oncogenic functions or activity under conditions in which the wild-type gene is inactive. A classic example of oncogene activation by chromosomal translocations in cancer is observed in chronic myelogenous leukaemia, which is initiated by a reciprocal t(9;22) chromosomal translocation that fuses the ABL proto-oncogene to the BCR gene [109, 110]. The fusion gene encodes an oncogenic BCR/ABL fusion protein with enhanced tyrosine kinase activity. All leukaemic cells carry this chromosomal alteration, which is why inhibition of the excessive tyrosine kinase activity of the fusion protein induces complete remission in most patients [111, 112]. An additional example of oncogene activation by chromosomal translocation is the t(11;14) translocation that juxtaposes cyclin D1 (CCND1) and immunoglobulin enhancer elements and is characteristic of mantle-cell lymphoma [113, 114].

Chromosomal translocations can also activate transcription-factors in cancers. For example, in Ewing’s sarcoma the EWS gene is fused with one of a number of genes, leading to altered transcriptional activity of the fused proteins [115]. The EWS protein is an RNA-binding molecule that, when fused to a heterologous DNA binding domain, can greatly stimulate gene transcription [104]. Prostate carcinomas carry translocations of the TMPR552 gene that fuse with and activate ERG1 or ETV1. These genes are members of the ETS family of transcriptional factors, which can activate or repress genes involved in cellular proliferation, differentiation, and apoptosis. The fusion of TMPR552 with an ETS-related gene creates a fusion protein that increases proliferation and inhibits apoptosis of cells in the prostate gland, thereby facilitating their transformation into cancer cells [116].

#### Genomic Amplifications

Oncogene activation by genomic amplification, which usually occurs during tumour progression, is seen in the members of different oncogene families including MYC, CCND, EGFR, and FOS. MYC is amplified in small-cell lung cancer, breast cancer, esophageal cancer, cervical cancer, ovarian cancer, and head and neck cancer. c-MYC gene is also amplified in a small subset of osteosarcomas [78, 117] and its product was found to be overexpressed more frequently in relapsed and metastatic disease [118]. CCND1 amplification occurs in breast, esophageal, hepatocellular, and head and neck cancer. EGFR (also called HER2/neu and ERBB2) is amplified in glioblastoma and head and neck cancer. Amplification of EGFR in breast cancer correlates with a poor prognosis [119]. Amplification is also seen in the dihydrofolate reductase gene (DHFR) in methotrexate-resistant acute lymphoblastic leukaemia [93, 94]. Amplification of DHFR is accompanied by cytogenetic alterations that mirror amplification of oncogenes [120, 121]. The amplified DNA segment usually involves several hundred kilobases and can contain many genes.

The c-FOS oncogene is overexpressed in a number of tumours including osteosarcoma breast carcinoma, cervical cancer, ovarian cancer and lung cancer [122]. In osteosarcoma c-FOS is amplified leading to its over expression [118] however in the other tumour types, the role of amplification in the gene overexpression is unresolved. It was isolated as the cellular homologue of the v-FOS gene found in the osteosarcoma inducing FBR- and FBJ-murine sarcoma viruses [123]. c-Fos oncogene is a transcription factor on chromosome 6q21 and its activation induces transformation in cultured cells [124]. Moreover, when the viral homologue v-FOS was injected into mice, osteosarcoma formation was enhanced [125]. Expression of c-FOS has been shown to be highly elevated in 60% of osteosarcoma samples [87]. Elevated expression of c-FOS was correlated with high-grade more frequently than with low-grade osteosarcoma [87]. Overexpression of c-FOS have been observed more often in patients who developed metastases than those who remained metastases free [118].

While activation of specific oncogenes and disruptions of individual tumour suppressors alter the tumour phenotype in a specific manner, cumulative effects of such changes may be more apparent in tumours with higher levels of genomic instability. Most cancers have an abnormal chromosomal content characterized by changes in chromosomal structure and number. Chromosomal aberrations are generally more numerous in malignant tumours than in benign ones, and the karyotypic complexity and cellular heterogeneity observed is often associated with poor prognosis.

### Genomic Instability

Genomic instability refers to a series of chromosomal changes occurring at an accelerated rate in cell populations derived from the same ancestral precursor [3]. It is a general term to describe the overall processes that increase the rate of mutation, thus enabling cells to develop new and aggressive phenotypes to adapt to the changing selection pressures [3]. Genomic instability is generally classified into two major types: microsatellite instability (MIN), and chromosomal instability (CIN) [126]. MIN involves simple DNA base changes that occur due to defects in the DNA repair processes including base excision repair, mismatch repair and nucleotide excision repair [127, 128]. CIN, on the other hand, is characterized by grossly abnormal karyotypes, featuring both structural and numerical chromosome abnormalities [129]. MIN and CIN mechanisms are generally found to be mutually exclusive and to produce different phenotypes [129] although recent findings suggest there may be some overlap in these two pathways [130].

Genome-wide and gene-specific epigenetic changes may presumably have similar effects on chromosomal structure and number in the affected cells. One of the challenges facing cancer researchers today is to understand how cancer cells acquire genomes with such a high degree genomic instability, and to determine in what way the genome and epigenome of cancer maybe interacting to facilitate the occurrence of such instability. In the coming section we will review some of the basic knowledge of epigenetic processes of DNA methylation and its relevance in cancer with special focus on the relationship between epigenetic and genetic mechanisms in tumours.

## DNA METHYLATION

DNA methylation is an epigenetic process involved in regulation of many cellular processes including gene expression, imprinting, X chromosome inactivation, silencing of retroviral and transposable DNA elements, and chromatin organization. DNA methylation refers to the addition of a methyl group to the fifth position of a cytosine. Methylated cytosines are present in the DNA of all vertebrates and flowering plants, some fungal, invertebrate and protist taxa, many bacterial species, and is common to all large genome eukaryotes [131]. In the following sections we will review our current knowledge of proteins involved in establishment and maintenance of DNA methylation including DNA methyltransferases and methyl binding proteins, normal functions of DNA methylation, changes in DNA methylation machinery related to human disease, and DNA methylation in human cancer.

### The DNA Methyltransferases (DNMTs)

Cytosine methylation is mediated by proteins called DNA (cytosine-5) methyltransferases. It has been more than 30 years since the prediction of two different classes of DNA methyltransferases (DNMTs) [132, 133]: maintenance DNMTs that preserve the patterns of DNA methylation during cell division by specifically acting on the hemimethylated DNA produced by semiconservative replication, and *de novo* DNMTs that establish methylation patterns on specific sequences early in development. Five DNA methyltransferases whose functions have been characterized have been identified including: DNMT1, DNMT2, DNMT3a, DNMT3b, and DNMT3L. 

The first DNA methyltransferase enzyme to be purified was DNMT1 [134]. Subsequent experiments showed that DNMT1 has an increased rate of methylation on hemimethylated DNA compared to the unmethylated DNA [135]. This preference of DNMT1 for hemimethylated DNA caused it to be assigned function of maintenance methyltransferase, although it is the only methyltransferase to be purified and cloned based on its *de novo* methylase property. The second DNMT to be identified based on its similarity to the bacterial type II cytosine-5’ methyltransferase was DNMT2 [136]. Although DNMT2 is the most strongly conserved and most widely distributed [131], it remains as the most enigmatic DNMT because it shows no detectable DNA methyltransferase activity [137]. Two additional DNA methyltransferases were identified from the EST databases and due to their similarity were named DNMT3a and DNMT3b [136, 138]. Mouse DNMT3a and DNMT3b were shown to *de novo* methylate DNA in human cells, with each enzyme having specific preferences for the different DNA regions [139]. The methyltransferase, DNMT3L, is a protein that is homologous to the other DNMT3s but has no catalytic activity. Instead, DNMT3L was shown to assist the *de novo* methyltransferases in establishment of maternal imprinting patterns during development [140, 141].

### The Methyl-Binding Proteins

In addition to the DNMTs, proteins which recognize and bind to methylated DNA play an important role in chromatin regulation. These include methyl-CpG-binding 2 protein (MeCP2) and methyl-binding domain proteins (MBD1 through 5).

MeCP2 was the first protein which was found to specifically bind methylated CpGs [142, 143]. It was also shown that MeCP2 can bind chromatin and that it plays a role in transcriptional repression of genes with methylated promoters through interactions with mSin3a/HDAC (histone deacetylase) chromatin remodelling complexes [144, 145]. Deletion of MeCP2 gene in mice results in embryonic lethality [146]. MBD1 through 4 were identified by database screening using the methyl-binding domain (MBD) of MeCP2 [147]. MBD1 was found to act as a transcriptional repressor in an HDAC-dependant manner, but does not associate with MeCP2-related complexes [148]. MBD2 binds methylated promoters and represses transcription through HDAC-related complexes, similarly to MeCP2 [149, 150]. MBD3 is a component of a Mi-2/NuRD transcriptional co-repressor complex [151, 152]. MBD4 is a glycosylase which removes mismatched thymine or uracil opposite CpG dinucleotides [153]. MBD5 was identified using a yeast-two-hybrid screen using p120 catenin and was named Kiaso [154]. It acts as a transcriptional repressor, but unlike MeCP2 and MBD2 which bind a single symmetrically methylated CpG, Kiaso requires at least a pair of CpGs [155].

### Normal Functions of DNA Methylation

The establishment and maintenance of proper DNA methylation patterns is essential for growth and development, as well as many cellular processes such as imprinting, X chromosome inactivation, silencing of retroviral and transposable DNA elements, and chromatin organization.

DNMT gene targeting experiments have shown the necessity of these methyltransferases in embryonic development. For example, Dnmt1 deficient mice displayed embryonic lethal phenotype with reduced size and gross morphological abnormalities at day 10.5 d.p.c. [156]. Also, maintenance enzyme Dnmt3a and Dnmt3b double knockouts showed a premature lethal phenotype at 4 weeks of age and at the late embryonic stages respectively, as well as lack a of *de novo* DNA methylation [157].

Further evidence that DNA methylation is essential for development comes from imprinting studies. Imprinting is a process in which a number of developmentally-important genes are marked for specific expression from either paternal or maternal chromosomes during oocyte and sperm production [158]. While differential DNA methylation plays a role in this process, disruption of DNA methylation in DNA methyltransferase-deficient mice has been shown to result in deregulation of expression in a number of imprinted genes including H19, insulin-like growth factor 2 (Igf-2), and Igf-2 receptor (Igf-2r) [159].

An additional important role of DNA methylation relates to the X chromosome. First recognized in 1948 by Murray Barr, the “Barr body” was later shown to be a product of X-chromosome inactivation. Inactivation of one of the two copies of X-chromosomes in mammalian females ensures gene dosage compensation to mammalian males, which carry a single X-chromosome [160]. DNA methylation is essential for appropriate X-inactivation, along with other mechanisms including histone modifications [161]. 

In addition to silencing the X-chromosome, most of the methylated cytosines in human DNA reside in, and repress, transposable and retroviral elements. It was shown that the loss of DNA methylation in Dnmt1-/- mice resulted in demethylation and transcriptional activation of intracisternal type A particle (IAP) retroviruses in developing mouse embryos [162]. Methylation of these sequences which include Alu, and short and long interspersed nuclear sequences (SINE and LINE), also increases the rate of their mutational inactivation *via *cytosine to thymine transitions. As such, DNA methylation is thought to have evolved as a mechanism related to containment of these potentially harmful genomic elements [163].

This broad functionality of DNA methylation in mammalian cells validates the need for stringent regulation of this process. Aberrant DNA methylation results in disruption of genomic and gene specific methylation profiles and ultimately leads to many human diseases.

### DNA Methylation and Human Disease

Many human diseases have been linked to aberrant DNA methylation or mutations in the DNA methylation machinery. The most common mutation-related diseases are Rett syndrome and ICF syndrome, as well as disorders related to aberrant imprinting such as Angelman syndrome, Prader-Willi syndrome, and Beckwith-Wiedemann syndrome.

Rett syndrome is caused by a dominant mutation in the X-linked methyl-CpG-binding MeCP2 gene [164]. It is thought that brain-derived neutrophic factor (BDFN), which is a specific target for MeCP2 [165], is one of the genes that is deregulated due to the loss of transcriptional repression of its target genes *via *mutant MeCP2. Immunodeficiency, Centromere instability, and Facial anomalies (ICF) syndrome which is caused by mutations in DNMT3b [166], is characterized by centromeric instability of chromosomes 1, 9 and 16, which is associated with abnormal hypomethylation of CpG sites within pericentromeric satellite regions. Expression profiling of ICF patients identified over 30 genes specific for lymphocyte signalling that are deregulated in this immune system disorder as a result of a DNMT3b mutation [167]. The two imprinting disorders Angelman and Prader-Willi syndrome are associated with the loss or mutation of the common imprinting centre at the 15q11-q13 region that contains 4 paternally and 2 maternally expressed imprinted genes [168]. Angelman syndrome is characterized by the loss of maternal contribution of 15q11-q13 or by paternal uniparental disomy (UPD) [169], while PWS results from deletion of paternal 15q11-q13 or maternal UPD [170]. An additional imprinting disorder, Beckwith-Weidemann syndrome, can be caused by mutations in the CDKN1C gene, alterations in the 11p15 region that result from parental UPD, or by deletions resulting in a loss of imprinting in this region [168, 171, 172].

There is also emerging evidence of links between aberrant DNA methylation and many other human diseases and conditions including neurological and cardiovascular disorders, imprinting and paediatric syndromes, reproductive problems, and aging [173]. The most compelling evidence of DNA methylation disorders and human pathogenesis is evident in human cancer. 

### DNA Methylation and Human Cancer

Malignant cells show major disruptions in their DNA methylation profiles which manifest as hypermethylation of gene promoters, global hypomethylation, and increased rate of mutation at methylated CpG dinucleotides.

Hypermethylation of CpG islands in gene promoters has been the most extensively studied area of research of DNA methylation in cancer. In contrast to the rest of the human genome, which is largely deprived of CpG dinucleotides, approximately half of all human genes promoters are associated with CpG-enriched regions (0.5-5 kb) called CpG islands [174]. In normal cells, the majority of the CpG islands are not methylated, allowing for transcription of their associated genes. In cancer cells, hypermethylation of these regions results in the loss of gene expression [7]. To date, many genes with aberrant promoter hypermethylation have been identified in essentially all forms of cancer. Some of these susceptible genes include cell cycle regulators (P16^INK4a^, P15^INK4a^, RB, P14^ARF^), DNA repair genes (BRCA1, MGMT, MLH1), genes associated with apoptosis (DAPK, TMS1), hormonal regulation (ER) detoxification (GSTP1), metastasis (E-cadherin, CD-44), angiogenesis (TSP-1, TIMP-3) and many others [175, 176]. Although some genes such as p16 are methylated in many cancers, other genes are methylated in specific types of cancer [176]. An example is GSTP1 which is hypermethylated in over 90% of prostate cancers, but is largely unmethylated in acute leukaemia [177, 178]. 

The second form of DNA methylation defect in many types of cancer is genomic hypomethylation [179]. It is common in both solid tumours such as prostate cancer [180], hepatocellular cancer [181], cervical cancer [182], as well as in haematologic cancers such as B-cell chronic lymphocytic leukaemia [183]. Aberrant hypomethylation has been hypothesized to contribute to cancer progression by activating oncogenes such as H-RAS [81], BORIS/CTCFL [184], FGFR1 [97], c-MYC [185], or by retrotransposon activation [186, 187] or by increasing chromosome instability as in ICF syndrome [188]. 

Finally, DNA methylation is also linked to tumourigenesis through mutational gene inactivation. Deamination of a methylated cytosine in the CpG dinucleotide causes a cytosine to thymidine transitional mutations in genes such as the tumour suppressor P53 and the human LDL receptor [189]. Analysis of DNA methylation in the coding regions of BRCA1, RB1, and NF1 showed prevalent CpG methylation, including those CpGs at mutational hotspots of these genes [190-192]. 

## INTERPLAY OF EPIGENETIC AND GENETIC CHANGES IN CANCER

While much of our understanding of cancer as a genetic or epigenetic disease comes from studies focusing on either mechanism specifically, some of the early studies and particularly many of the new developments in the field are revealing the importance of the interplay of both genetic and epigenetic changes in tumours. Genomic imbalance and DNA methylation have been shown to disrupt normal gene expression and gene dosage, while disruptions of DNA methylation profiles may play a role in genomic instability especially at repeat-rich sequences.

It has been nearly a decade since Knudson’s two-hit hypothesis was expanded to encompass aberrant DNA methylation as an alternative inactivation mechanism for disruption of tumour suppressor gene expression [193]. Many studies at that time focused on promoter DNA hypermethylation as a means of inducing loss of tumour suppressor gene function. (Table **1**) shows a list of some of the most common tumour suppressors that have been identified to date, whose expression has been shown to be affected by both genetic aberrations as well as promoter DNA hypermethylation. Hypermethylation of tumour suppressor genes in cancer has been reviewed extensively and reader should refer to the following references for more information [6, 7, 194-196]. In addition to promoter DNA hypermethylation of tumour suppressors, promoter DNA hypomethylation of oncogenes can affect gene expression similarly to a genomic amplification or oncogenic translocation. 

The first identified cancer-related aberrant methylation of a gene promoter was hypomethylation of c-Ha-RAS and c-Ki-RAS oncogenes in primary human carcinomas in 1983 [81]. Recently, FGFR1 amplification in rhabdomyosarcomas was associated with both hypomethylation of its upstream CpG island as well as overexpression of this gene and induction of its downstream targets [97]. c-MYC is another oncogene that was shown to be hypomethylated in acute leukaemia derived from myelodysplastic syndromes [185]. In a recent study it was shown that hypomethylation of the LINE 1 retrotransposon, as well as amplification of MYC can be used to predict tumour stage in prostate cancer [197]. Genome wide functional approach using Decitabine exposure identified oncogenes ELK1, FRAT2, r-RAS, RHOB, and RHO6, as gene candidates that are silenced by DNA methylation in normal stomach mucosa but are activated by DNA demethylation in a subset of gastric cancers. Authors further showed that demethylation of specific CpG sites within the first intron of r-RAS causes its activation in more than half of gastric cancers [82]. Another study showed drastic hypomethylation and overexpression of Ha-RAS gene promoter in a mouse carcinogenesis model which authors concluded supports the hypothesis that tumour promotion involves instability of the epigenome, providing an environment where changes in the methylation status of specific regions of the genome accumulate progressively and contribute to the clonal expansion of initiated cells that leads to tumour formation [80]. Similarly, another group showed hypomethylation of Ha-Ras gene promoter in response to a high arsenic diet of a mouse carcinogenesis model [83]. Environmental exposure studies have shown that carcinogenic chemicals such as arsenic and selenium may cause genomic DNA hypomethylation during the process of detoxification of these metals [198, 199]. Exposures to benzo(a)pyrene carcinogen were shown to induce genome wide DNA and repeat-specific hypomethylation, and histone hyperacetylation in a breast cancer model [200-202]. Therefore, cumulative evidence indicates that much like hypermethylation of tumour suppressors, hypomethylation of oncogenes plays a critical role in tumour evolution, and that genomic hypomethylation is linked to process of carcinogenesis in general.

These findings suggest that DNA methylation provides an additional “layer” of control of gene expression during the process of tumourigenesis. Furthermore, there is now increasing evidence that methylation may be more directly involved in the process of genomic destabilization and human cancer. In addition to genomic instability, global DNA hypomethylation is evident in many cancers including prostate [180], hepatocellular [181], cervical [182], B-cell chronic lymphocytic leukaemia [183], bladder [203] and liver cancer [204]. Decreased levels of global DNA methylation appear to be indicative of increased pathologic grade in many malignancies including those of breast, cervix and brain [179]. 

The hypomethylation in cancer cells of retrotransposons such as LINE elements can cause their transcriptional activation [205]. Such active transposons can disrupt other genes through mutational insertions. For example, an active L1 LINE element was shown to disrupt the c-MYC and APC genes in breast and colon cancer respectively [206]. Recent genomic array profiling of DNA methylation in lung cancer identified extensive DNA hypomethylation in tumours occurs specifically at repetitive sequences, including short and long interspersed nuclear elements and LTR elements, segmental duplications, and subtelomeric regions [207]. Hypomethylation of repeat elements in cancer is particularly intriguing given the causative roles such sequences play in generation and propagation of genomic instability [208]. Another study involving genome-wide characterization of hypomethylated sites in human tissues and breast cancer cell lines has also identified megabase-sized hypomethylated zones that are associated with large genes, fragile sites, evolutionary breakpoints, chromosomal rearrangement breakpoints, and tumour suppressor genes [209]. Analysis of DNA methylation of genomic DNA repetitive elements (LINE1, Alu, Satellite-alpha and Satellite-2) during the progression of CML from chronic phase to blast crisis showed that chronic-phase CML samples were significantly more hypomethylated for all repetitive sequences compared with normal samples and a more profound level of hypomethylation was observed among blast crisis samples compared to chronic phase samples [210]. Another group showed a strong correlation between LINE1 hypomethylation and amplification of 8q chromosome arm in prostate cancer [211]. Genomic gain of 1q arm in hepatocellular carcinoma was also shown to be significantly correlated to hypomethylation of centromeric heterochromatin satellite 2 DNA, at the 1q12 fragile site [212]. The major breakpoint cluster region (M-BCR) that is involved transformation of CML from the chronic to the blastic phase was shown to undergo various levels of hypomethylation related to lymphoid-crisis patients studied in blastic phase. Therefore, in addition to reactivation of oncogene expression, global DNA hypomethylation plays a role in reactivation of repetitive elements and subsequent genome destabilization. 

## EPI/GENETIC MODEL OF TUMOUR EVOLUTION

It has become evident that there is a strong link between epigenetic disruptions of DNA methylation and regions of genomic instability in human cancer. While early studies focused primarily on DNA hypermethylation of tumour suppressors, an increasing body of evidence indicate that hypomethylation of oncogenes represents another epigenetic event common in tumourigenesis. In addition to directly affecting gene expression, DNA methylation plays an important role in maintenance of genomic stability, particularly by repressing repetitive genomic elements, disruption of which is closely related to genomic instability and chromosomal aberrations. Fig. (**1**) presents our model of tumour progression in which underlying genetic and epigenetic changes drive tumour evolution by disrupting both normal gene expression and gene dosage, while concurrently increasing genomic instability, and as a result providing selective advantage to newly formed tumour cells.

This model further adds to Jones and Laird model [193] which primarily focused on genetic and DNA hypermethylation-mediated tumour suppressor inactivation, by including both hypomethylation of oncogenes and hypomethylation of repeat elements as key events in genomic destabilization and tumour evolution. Our model suggests that normal gene expression profiles depend on both genomic content and epigenetic DNA methylation profiles, which coordinately ensure appropriate levels of gene expression in normal cells. Furthermore, normal patterns of genomic DNA methylation play a crucial role in repression and condensation of inactive and repetitive elements thereby ensuring appropriate chromatin conformation in these regions. When such elements are disrupted chromatin changes may lead to instability with consequent chromosomal and expression changes. During the evolution of tumour phenotype disruptions of both genetic and epigenetic DNA methylation profiles, such as loss and/or hypermethylation of tumour suppressors, gain and/or hypomethylation of oncogenes, as well as increased genomic instability related to disruption of genomic and epigenomic profiles will have selective advantages in the population of tumour cells. This process can drive the evolution of tumour cells, and provide selective advantages to cells with most favourable gene expression phenotypes, which as we suggest will be a result of a dual “layer” control through both genetic changes and DNA methylation. 

In addition to genetic and DNA methylation changes, many other factors may play a role in genomic stability and regulation of gene expression including chromatin-related factors such as histone tail modifications and ATP-dependent chromatin remodelling complexes, transcriptional factor binding, non-coding regulatory RNA molecules, tissue microenvironment and others. The unique characteristic of both genetic changes and DNA methylation is that they are heritable. By definition, heritability is a requirement for the concept of tumour evolution. As we understand more about many other factors that may influence tumour evolution and the potential heritability of such mechanisms through the cell cycle, this model may be augmented to include those changes. 

Another important feature of many solid and haematologic cancers is the remarkable inter- and intra-tumour heterogeneity in both genetic changes and DNA methylation profiles. Since changes in gene expression and subsequent disruption of protein network interactions allow for and are a major driving force in tumour evolution, underlying regulatory mechanisms of gene expression must play an important role. Complimentary and sometimes mutually exclusive genetic and epigenetic roles may provide a dual “layer” of control of gene expression that neither mechanism alone can address independently. Fig. (**2**) illustrates how dual “layer” genetic and DNA methylation changes can provide selective advantages with greater plasticity for the tumour evolution, whilst simultaneously increasing tumour heterogeneity and proliferative/selective advantages for the most adaptive cellular subtypes.

## IMPLICATIONS OF EPI/GENETIC MODEL OF TUMOUR EVOLUTION

Our ability to identify diagnostic, prognostic, and therapeutic genetic changes in majority of tumours has been very limited. Focusing solely on genetic changes that may underlay changes in gene expression and therefore influence tumour evolution may have biased our predictive capability to a single “layer” of at least dual-layered levels of heritable control of gene expression. Focusing solely on epigenetic changes in DNA methylation may suffer from the same bias. Therefore, in order to increase our predictive power in the studies of mechanisms underlying heritable changes in gene expression profiles and consequently tumour evolution, there needs to be an emphasis on the integration of genetic and epigenetic information in relation to gene expression changes on the genomic level.

Biotechnological revolution in the past decade has for the first time allowed for genome wide screening of expression profiles, genomic changes, and most recently epigenomic changes in DNA methylation, allowing for some remarkable improvements in our understanding of cancer genotype, epigenotype, and gene expression phenotype. With the development of biologist-friendly integrative software tools we may for the first time be able to increase our predictive powers for detection of mechanisms responsible for the acquisition of cancer phenotype and tumour evolution.

While genetic changes are permanent and virtually irreversible, epigenetic changes in DNA may be reversed. In a clinical setting DNA methylation represents a very attractive target for the development and implementation of new therapeutic approaches. Much effort is spent on identification of potential chemotherapeutics that may modulate DNA methylation, and one of them, Decitabine, is currently being used in clinic. Many clinical trials are ongoing and epigenetic therapy has recently been approved by the United States Food and Drug Administration (US FDA) for the use in the treatment of myelodysplastic syndrome (MDS) and primary cutaneous T-cell lymphoma (CTCL). Identification of epigenetically-deregulated gene pathways in the context of tumour-specific genetic changes may provide a therapeutic target at the gene transcription level, augmenting our efforts of targeting the genetic disruptions at the level of protein. However, given the non-specific nature of current epigenetic drugs that may target and reactivate oncogenes in addition to the tumour suppressors, caution and further investigation of gene-specific methylation modifying drugs are warranted.

In addition to being reversible, epigenetic changes in DNA methylation are environmentally responsive in toxicological, nutritional, and psychosocial sense [213]. As such, integration of epigenetic information to the genetic profiles of cancer may for the first time allow us to gain a more complete understanding of genetic basis and environmental influences in the etiology of this complex disease.

## Figures and Tables

**Fig. (1):**
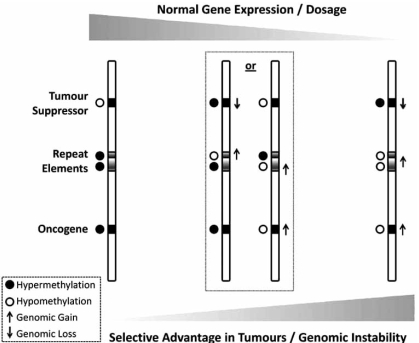
Acquisition of genetic and epigenetic changes disrupts normal gene expression and provides selective advantage to cancer cells.

**Fig. (2):**
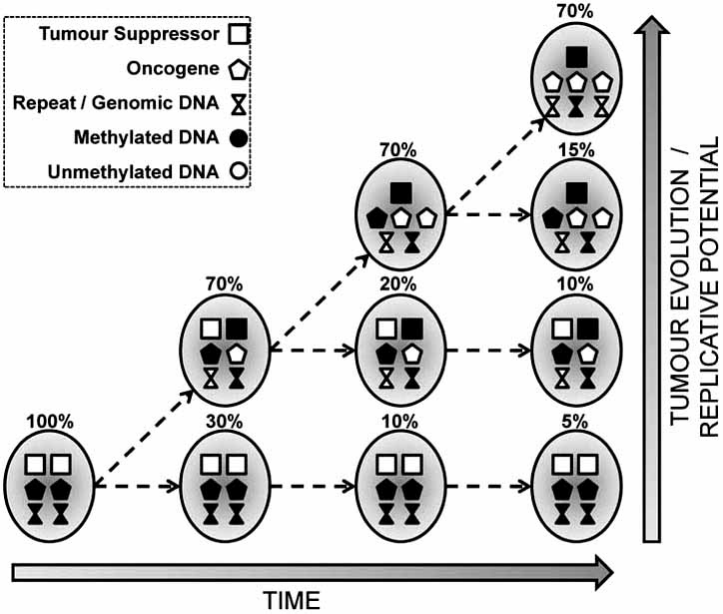
Co-evolution of genetic and epigenetic changes creates tumour heterogeneity, increases replicative potential and drives tumour evolution.

**Table 1.** Genetic and DNA Methylation Changes in Common Cancer Related Genes

Cancer-Related Genes	Genetic Change	DNA Methylation	Major Tumour Sites / Types	References
**Tumour Suppressor Genes**				
**P53**	mutations, allelic loss and LOH	hypermethylation	breast, brain, sarcomas including osteosarcoma	[18, 29-32]
**RB**	mutations, allelic loss and deletions	hypermethylation	retinoblastoma	[33-42]
**APC**	mutations	hypermethylation	colon, thyroid, intestine, stomach	[43-46]
**MSH2 & MLH1**	mutations	hypermethylation	colon, uterus	[47-49]
**WT1**	allelic loss	hypermethylation	Wilms' tumors	[50-52]
**PTEN**	mutations and LOH	hypermethylation	glioma, uterus	[53-55]
**P21 (CDKN1A)**	mutations	hypermethylation	squamous cell carcinoma, melanoma	[56, 57]
**INK4A, ARF**	deletions	hypermethylation	melanoma, osteosarcoma	[23, 28, 58, 59]
**ATM**	mutations	hypermethylation	leukemia, lymphoma, brain	[60-62]
**BRCA1, BRCA2**	mutations, LOH, deletions, allelic loss	hypermethylation	breast, ovary	[19, 63-69]
**REQL4**	mutations	?	skin, osteosarcoma	[70, 71]
**Oncogenes**				
**EWS/FLI-1**	translocations	?	Ewing’s sarcoma	[72, 73]
**BCR/ABL**	translocations	?	CML	[74, 75]
**MYC**	amplifications	hypomethylation	lung, breast, colorectal, head/neck, osteosarcoma	[76-79]
**RAS and RAS-family**	mutations	hypomethylation	lung, colon, pancreas, AML	[80-85]
**CDK4**	amplification	?	osteosarcoma	[23]
**FOS**	amplifications	hypomethylation	osteosarcoma	[86, 87]
**EGFR (ERBB2)**	amplifications	hypermethylation	glioblastoma, head/neck, osteosarcoma	[88-91]
**PAX3/FKHR**	translocations	?	alveolar, rhabdomyosarcoma	[92]
**DHFR**	amplifications	?	ALL	[93, 94]
**MET**	mutant allele duplications	?	papillary renal carcinoma	[95, 96]
**FGFR1**	amplification	hypomethylation	rhabdomyosarcoma	[97]
**RET**	mutant allele duplications	?	endocrine neoplasia	[98-100]
**CCND1**	amplifications and translocations	?	breast, esophageal, hepatocellular,head/neck, mantle-cell lymphoma	[76, 101-103]
**TMPR552/ERG1 or TMPR552/ETV1**	translocations	?	prostate cancer	[104]

## ABBREVIATIONS

LOH: = Loss of heterozygosity
MIN: = Microsatellite instability
CIN: = Chromosomal instability
DNMT: = DNA methyltransferase
MBD: = Methyl-binding domain
MECP2: = Methyl-CpG-binding 2 protein
HDAC: = Histone deacetylase
LINE: = Long interspersed nuclear element
SINE: = Short interspersed nuclear element
IAP: = Intracisternal type A particle
UPD: = Uniparental disomy
